# Utilization of an Eilat Virus-Based Chimera for Serological Detection of Chikungunya Infection

**DOI:** 10.1371/journal.pntd.0004119

**Published:** 2015-10-22

**Authors:** Jesse H. Erasmus, James Needham, Syamal Raychaudhuri, Michael S. Diamond, David W. C. Beasley, Stan Morkowski, Henrik Salje, Ildefonso Fernandez Salas, Dal Young Kim, Ilya Frolov, Farooq Nasar, Scott C. Weaver

**Affiliations:** 1 Institute for Translational Sciences, University of Texas Medical Branch, Galveston, Texas, United States of America; 2 Center for Tropical Diseases, University of Texas Medical Branch, Galveston, Texas, United States of America; 3 InBios International, Inc., Seattle, Washington, United States of America; 4 Departments of Medicine, Molecular Microbiology, Pathology & Immunology, Washington University School of Medicine, St. Louis, Missouri, United States of America; 5 Institute for Human Infections and Immunity, Department of Microbiology and Immunology, University of Texas Medical Branch, Galveston, Texas, United States of America; 6 Center for Biodefense and Emerging Infectious Diseases, and Sealy Center for Vaccine Development, University of Texas Medical Branch, Galveston, Texas, United States of America; 7 Johns Hopkins Bloomberg School of Public Health, Baltimore, Maryland, United States of America; 8 Pasteur Institute, Paris, France; 9 Centro Regional de Investigación en Salud Publica INSP. Tapachula, Chiapas, Mexico; 10 Department of Microbiology, University of Alabama at Birmingham, Birmingham, Alabama, United States of America; 11 Virology Division, United States Army Medical Research Institute of Infectious Diseases, Frederick, Maryland, United States of America; Centers for Disease Control and Prevention, UNITED STATES

## Abstract

In December of 2013, chikungunya virus (CHIKV), an alphavirus in the family *Togaviridae*, was introduced to the island of Saint Martin in the Caribbean, resulting in the first autochthonous cases reported in the Americas. As of January 2015, local and imported CHIKV has been reported in 50 American countries with over 1.1 million suspected cases. CHIKV causes a severe arthralgic disease for which there are no approved vaccines or therapeutics. Furthermore, the lack of a commercially available, sensitive, and affordable diagnostic assay limits surveillance and control efforts. To address this issue, we utilized an insect-specific alphavirus, Eilat virus (EILV), to develop a diagnostic antigen that does not require biosafety containment facilities to produce. We demonstrated that EILV/CHIKV replicates to high titers in insect cells and can be applied directly in enzyme-linked immunosorbent assays without inactivation, resulting in highly sensitive detection of recent and past CHIKV infection, and outperforming traditional antigen preparations.

## Author Summary

We have developed an innovative approach to production of alphavirus antigens for use in diagnostic assays that results in reduced production complexity as well as improved sensitivity in application. By generating recombinant viruses that contain the structural protein genes of pathogenic alphaviruses and the nonstructural protein genes of an insect-specific alphavirus, Eilat virus, we have been able to produce insect-restricted viruses that are antigenically identical to their pathogenic counterparts. The insect-specific nature of these chimeric viruses yields an advantageous safety profile and allows for safe handling of the antigen at the bench top. Traditional antigens, produced from wild-type virus, require extensive processing, from growth at biosafety level 3 to concentration and inactivation, followed by lyophilization, which often compromises antigen reactivity and is financially costly. Furthermore, current inactivation methods are imperfect processes that have historically resulted in residual live virus and subsequent breach of containment when used in a diagnostic setting. Other approaches, such as recombinant antigens generated from viral particle subunits, are missing conformational epitopes and their application results in reduced sensitivity. Here we describe the development of a diagnostic assay using this technology for the detection of chikungunya infection in humans.

## Introduction

The genus *Alphavirus* in the family *Togaviridae* is comprised of small, enveloped viruses with single-stranded, positive-sense RNA genomes 11–12 kb in length [*1*]. The genus includes 31 recognized species classified into eleven complexes based on antigenic and/or genetic similarities [*2–4*], with most utilizing mosquitoes as vectors [*1–7*]. Mosquito-borne alphaviruses can infect mosquito species encompassing at least eight genera as well as many vertebrate taxa [*8–12*]. This ability to infect vertebrates and mosquitoes enables the maintenance of alphaviruses in endemic cycles with sporadic spillover events into human populations. Infections by Old World alphaviruses including chikungunya (CHIKV), o'nyong-nyong, Sindbis, and Ross River viruses can produce rash and debilitating arthralgia [*13*]. In contrast, New World alphaviruses such as western (WEEV), eastern, and Venezuelan equine encephalitis (VEEV) viruses can cause fatal encephalitis [*13*].

In 2004, CHIKV reemerged from Africa and spread to the Indian Ocean Basin, Asia, and Europe causing explosive epidemics affecting millions of people [*14–20*]. Chikungunya fever (CHIKF) is characterized by severe, debilitating, and often chronic arthralgia that can persist for years, resulting in major economic as well as public health impacts [*18,20–22*]. Additionally, CHIKF is not easily diagnosed due to the overlap in initial signs and symptoms with dengue, malaria and other acute febrile illnesses, as well as the lack of high quality, affordable, commercially-available diagnostic assays [*23,24*].

In October of 2013, CHIKV arrived in the Caribbean from Asia or Oceania and spread to South, Central and North America, resulting in over 1.1 million suspected cases as of February 2015 [Pan American Health Organization (PAHO) data]. Over 2,000 imported CHIKF cases have been detected in the U.S. and transmission in Florida has resulted in 11 autochthonous cases. Transmission in other parts of the U.S. is expected as CHIKV moves northward through Mexico. The large populations of both urban mosquito vectors (*Aedes aegypti* and *A*. *albopictus*) throughout most of the Americas, large populations of naive people, and its history in Southeast Asia indicate that CHIKV is likely to remain epidemic and endemic in the Americas for the foreseeable future [*25*].

Recently, a related alphavirus, Eilat virus (EILV), isolated from *Anopheles coustani* s.I. mosquitoes was described [*26*]. EILV groups phylogenetically within the mosquito-borne clade of alphaviruses as a sister to the WEE complex. Although EILV, like other alphaviruses, can replicate to high titers (>10^8^ PFU/mL) in mosquito cells, it is unable to replicate in vertebrate cells with blocks at both attachment/entry as well as viral RNA replication levels [*27*]. These unique EILV characteristics as well as the genetic tractability of alphaviruses provide an opportunity to safely develop antigens that, in contrast to traditional methods, do not require inactivation or expensive containment facilities to produce. Here, we report the development of an EILV/CHIKV chimera and its use as a diagnostic reagent to detect CHIKV infection in humans.

## Methods

### Cell culture

C7/10 cells (American Type Culture Collection, Rockville, MD), derived from *A*. *albopictus* mosquitoes, were propagated at 28°C with 5% CO_2_ in Dulbecco’s minimal essential medium (DMEM) containing 10% (V/V) fetal bovine serum (FBS), sodium pyruvate (1 mM), penicillin (100 U/mL), streptomycin (100 μg/mL), and 1% (v/v) tryptose phosphate broth (Sigma, St. Louis, MO).

### cDNA clone and rescue of infectious EILV/CHIKV

An infectious cDNA clone encoding the EILV genome was chimerized by replacing its structural polyprotein open reading frame with that of a human CHIKV isolate from the British Virgin Islands (strain-99659) [*26*, *28*]. The chimera was cloned and rescued as previously described [*27*]. Briefly, the CHIKV structural polyprotein open reading frame (ORF) was reverse transcribed from extracted RNA and PCR-amplified in three fragments between AvrII, Bsu36I, NcoI, and NotI restriction sites. These fragments were then digested and ligated into an infectious clone of EILV described previously [*26*] between AvrII and NotI sites, replacing the structural polyprotein ORF of EILV.

### Virus amplification

The EILV/CHIKV chimera was amplified by infection of C7/10 cells at a multiplicity (MOI) of 0.1 PFU/cell and at 48 hours post-infection (hpi), supernatants were harvested and clarified by centrifugation for 10 min at 3,000 × *g*. To generate serum-free EILV/CHIKV for use in indirect enzyme-linked immunosorbent assays (ELISAs), C7/10 monolayers were washed with PBS 6 hours post-infection, and VP-SFM medium (GIBCO, Grand Island, NY) containing penicillin (100 U/mL), streptomycin (100 μg/mL), and 1% (v/v) tryptose phosphate broth (Sigma) was added. Cells were incubated for an additional 42 hours and supernatants were harvested and stored at -80°C.

To compare EILV/CHIKV as antigen to a traditional antigen preparation, CHIKV (strain LR2006 OPY1), obtained from the World Reference Center for Emerging Viruses and Arboviruses (WRCEVA) at the University of Texas Medical Branch, was amplified by infection of confluent BHK-21 cells, and cell-lysate antigen (CLA) was prepared as previously described [*29*]. Briefly, infected cells were pelleted, washed with ice-cold borate saline buffer, and resuspended in an SDS/Triton X-100 buffer for sonication, followed by clarification by centrifugation and inactivation with 0.3% (v/v) β-propiolactone.

### Mouse antisera and antibodies

Mouse immune ascitic fluids (MIAFs) against CHIKV strain Ross T-36059, and Gamboa virus strain T-34953, a bunyavirus used as a negative control, were obtained from the WRCEVA. Mouse immune serum (MIS) raised against mosquito salivary proteins was generated as previously described [*30*]. Briefly, adult CD1 mice were naturally exposed to approximately 20 *A*. *albopictus* mosquito bites twice weekly for 4 weeks, at which point serum was collected for use in an ELISA. A mouse IgG monoclonal antibody (mAb) against CHIKV, CHK-175, was used as a quantitative benchmark [*31*].

### Human antisera and antibodies

For IgM ELISA positive controls, a panel of five IgM/ELISA- and PRNT-positive convalescent sera obtained from patients diagnosed by reverse transcriptase-PCR with CHIKV infection was used. Eight human serum samples positive for either dengue virus (DENV) or VEEV but negative for CHIKV by hemagglutination inhibition (HI) [*32*] were used as negative controls. To validate IgM ELISAs, a panel of acute serum samples collected from patients in Mexico with suspected CHIKV infection, based on clinical guidelines set forth by the Centers for Disease Control and Prevention (CDC) and PAHO [*33*], were characterized by plaque reduction neutralization test (PRNT) as described previously [*32*]. Thirty-two CHIKV PRNT-positive samples were then selected for comparative IgM ELISA.

For IgG ELISAs, 32 CHIKV PRNT-positive samples from Bangladesh, collected for a seroprevalence study from healthy individuals, were used. To determine a statistically robust cut-off value for human antibody-capture ELISAs, a panel of 34 human serum samples from Bangladesh, negative for CHIKV by PRNT, was utilized in addition to the 8 negative control samples described above.

### Indirect IgG ELISAs

Immulon 2HB 96-well plates (Fisher Scientific, Pittsburgh, PA) were coated with serum-free EILV/CHIKV culture supernatants diluted in PBS, to a final concentration of 5 x 10^4^ PFU per well or with CLA at a 1:400 dilution and incubated overnight at 4°C. These antigen dilutions were optimized in titration experiments against polyclonal sera. Plates were blocked with 100 μL of InBlock buffer (InBios, Inc., Seattle, WA) for 1 h at room temperature (RT) and washed 5 times with 300 μL of 0.1% Tween-20 in PBS using an automatic plate washer (BIO-RAD, Model 1575 ImmunoWash, Hercules, CA). Serum samples were diluted 1:100 in EB-C sample dilution buffer (SDB; InBios) and 2-fold serial dilutions were added to plates followed by incubation for 1 h at RT. Plates were washed as described above, and 50 μL of biotin-conjugated goat anti-mouse IgG (Jackson ImmunoResearch Laboratories, West Grove, PA) were added at a dilution of 1:10,000 in SDB and plates were incubated for 1 h at RT. Then, plates were washed, and 50 μL streptavidin-conjugated horseradish peroxidase (HRP) (Roche Diagnostics, Indianapolis, IN) were added at a dilution of 1:10,000 in SDB, and plates were incubated for 1 h at RT. Plates were washed and 75 μL of 3,3’,5,5’-tetramethylbenzidine substrate (TMB; Sigma) were added, incubated for 10 min at RT, and the reaction was stopped with 50 μL of 0.5M sulfuric acid. Absorbance values were read at 450 nm on a VERSAmax tunable microplate reader (Molecular Devices, Sunnyvale, CA).

### CHIKV IgM- and IgG-capture ELISAs

Human serum samples, diluted 1:100 in SDB, were added to human IgM- or IgG-capture 96-well microtiter plates (InBios) in 50 μL volumes. After 1 h incubation at 37°C, plates were washed as described above. Cell supernatant containing EILV/CHIKV diluted in 1% BSA in PBS to a concentration of 2.5X10^7^ PFU/well was then added and incubated for 1 h at 37°C.

A panel of eight anti-CHIKV mouse mAbs was tested independently or in combinations for activity in a capture ELISA. CHK-175 antibody produced the highest signal-to-noise ratio at an optimal concentration of 100 ng/well and was selected for further use as the detecting antibody. CHK-175 diluted in SDB to 100 ng/well was added and incubated for 1 h at 37°C. Plates were washed, 50 μL of goat anti-mouse IgG-HRP conjugated antibody (Southern Biotech, Birmingham, AL) diluted 1:5,000 in conjugate dilution buffer (InBios) were added, and plates were incubated for 1 h at 37°C. Plates were then washed, 75 μL of TMB was added, incubated for 10 min at RT, and the reaction was stopped by the addition of 50 μL 0.5M sulfuric acid. The absorbance values were read at 450 nm.

Commercially available anti-CHIKV IgM (ab177848, Lot: GR195090-3, Abcam, Cambridge, MA) and anti-CHIKV IgG Human ELISA Kits (ab177835, Lot: GR148047-1, Abcam) were used according to manufacturer’s instructions.

### Stability of EILV/CHIKV

The stability of EILV/CHIKV was assessed as described previously [*34*]. EILV/CHIKV was diluted 1:2 in PBS with 1% BSA, TRIS with 1% BSA, or SDB and incubated at 37°C. Antigen was sampled at 0, 3, 7, or 10 days post-incubation and the antigen stability was assessed in IgM-capture ELISA described above. The stability was compared to a 4°C control of EILV/CHIKV and percent of original activity was calculated.

### Animal safety study

Pregnant CD1 mice were obtained from Charles River (Wilmington, MA). Seven-day-old suckling mice were pooled and randomly distributed into three groups of 13 mice each and inoculated intracranially (IC) with either 8 log_10_PFU of EILV/CHIKV, 4 log_10_PFU of live-attenuated CHIKV strain 181/25 [*35*] as a positive control for replication and neurovirulence, or mock-infected with PBS. Animals were observed daily for 28 days and sacrificed when they became moribund. Three mice per group were sacrificed on 0, 3, 7, and 14 days post-infection and brain tissue was collected to determine infectious virus content via plaque assay on C7/10 cells as previously described [*26*].

### Statistical analysis

To compare the activities of EILV/CHIKV versus CLA in indirect ELISAs, and the effects of the EILV/CHIKV and Abcam methods on absorbance values and signal-to-noise ratios, a 2-way ANOVA using a Bonferroni post-hoc test was performed. To compare the effects of different buffer preparations on EILV/CHIKV stability and activity differences between high-, medium-, and low-positive serum groups tested using either EILV/CHIKV or Abcam ELISA, a 2-way ANOVA using Tukey’s multiple comparisons test was performed. All statistical calculations were performed in Prism 6 (GraphPad Software, Inc., La Jolla, CA). A cut-off absorbance value for CHIKV-positive human samples was calculated as 5 standard deviations above the mean absorbance value of 42 PRNT-negative human serum samples.

### Ethics statement

Deidentified, excess diagnostic serum samples, from Mexico and Bangladesh, were tested under a University of Texas Medical Branch institutional review board-approved protocol with no informed consent required. Animal studies were approved by the UTMB Institutional Animal Care and Use Committee under protocol 02-09-068. The euthanasia method employed was carbon dioxide exposure followed by decapitation.

## Results

### Evaluation of EILV/CHIKV as an antigen in indirect ELISA

**Fig 1:**
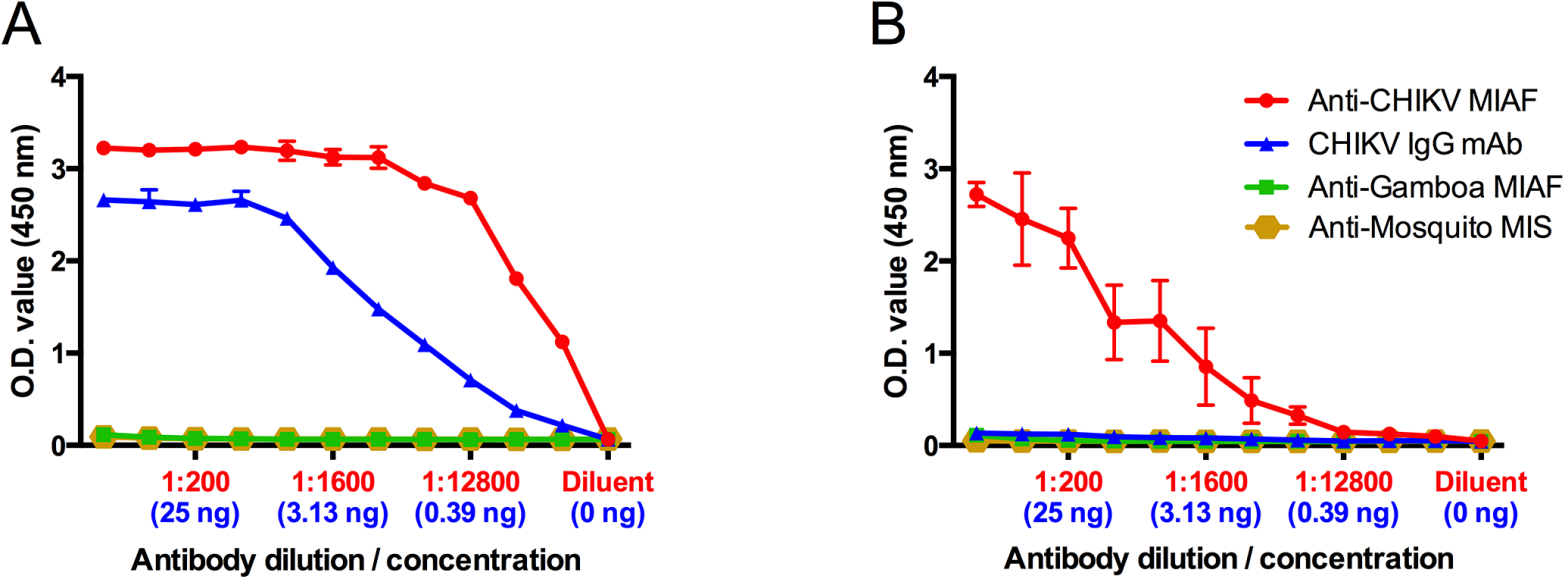
Indirect mouse anti-CHIKV IgG ELISAs utilizing either (A) EILV/CHIKV or (B) cell-lysate antigen to detect serially diluted polyclonal anti-CHIKV MIAF (measured over a range of serum dilutions, red) or monoclonal antibody CHK-175 (expressed in ng quantities, blue). MIAF against Gamboa virus and MIS against mosquito antigens were included as negative controls. Mean and standard deviation of 2 replicates are reported.

The utility of the EILV/CHIKV chimeric virus as a diagnostic antigen was first tested in an indirect ELISA. Plates were coated with EILV/CHIKV at 5 x 10^4^ PFU per well and antigen was detected by both polyclonal and monoclonal antibodies with dilutions from 1:50 to 1:51,200. EB-C sample dilution buffer, anti-Gamboa MIAF, and MIS raised against mosquito salivary proteins were utilized as negative controls. No cross-reactivity with mosquito-sensitized mouse sera was observed and optical density (OD) values of all of the negative controls were ≤ 0.07 (Fig 1A). In contrast, the mouse anti-CHIKV polyclonal antisera and CHK-175 monoclonal antibody readily detected antigen at all dilutions with OD values from 1.1–3.2 and 0.2–2.7, respectively. Signal-to-noise ratios ranged from ~16–46:1 and ~3–39:1 for polyclonal and monoclonal antibodies, respectively. As antigen was detected at all serum dilutions, an estimated quantitative sensitivity of the EILV/CHIKV ELISA was determined by titrating CHK-175 antibody from 100 μg/mL to 98 ng/mL. The lowest concentration of 98ng/mL was capable of detecting antigen, indicating a very high sensitivity of the assay.

In contrast to the results obtained with EILV/CHIKV, CLA could only be detected by the polyclonal sera (Fig 1B). In addition, the OD values obtained with polyclonal sera ranged from 0.1–2.7 and were significantly lower at all dilutions (p<0.05).

### Utilization of the EILV/CHIKV as antigen to detect human IgM and IgG in capture ELISA

**Fig 2:**
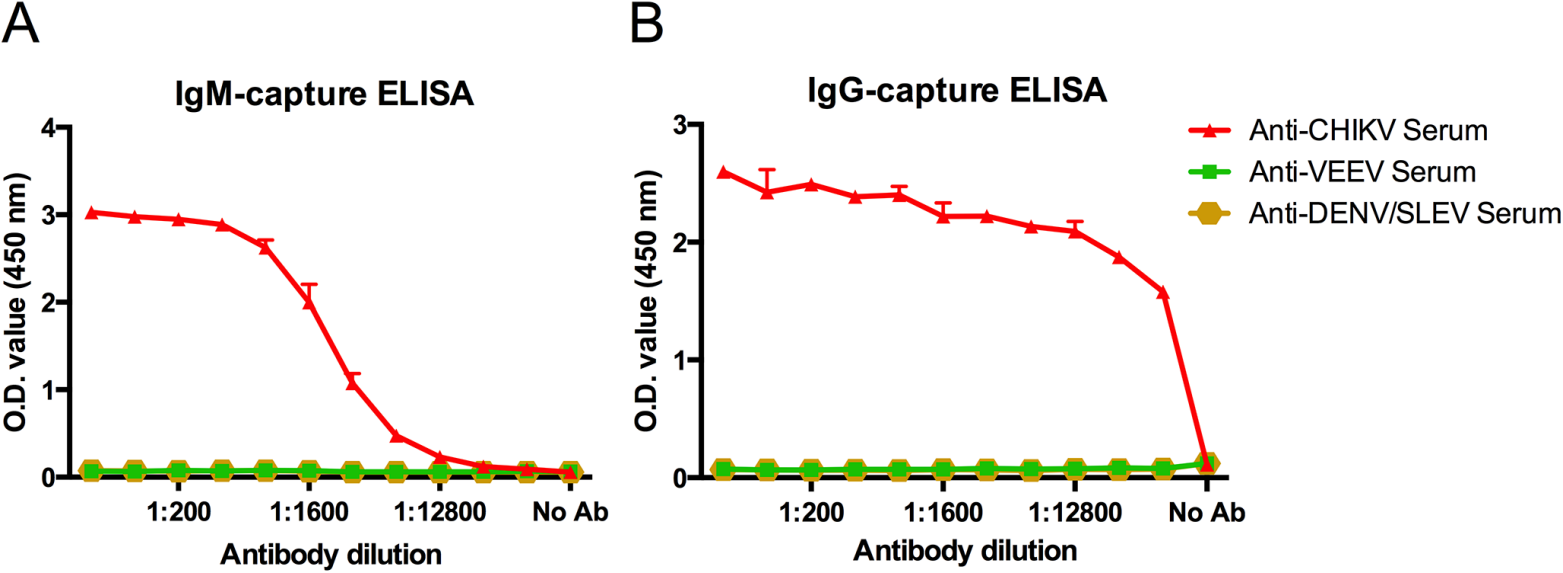
Human anti-CHIKV antibody-capture ELISAs utilizing EILV/CHIKV as antigen to detect either (A) IgM or (B) IgG antibodies (both measured over a range of serum dilutions, red). Human serum samples that were antibody-positive for either DENV or VEEV but negative for CHIKV by HI were included as negative controls. Mean and standard deviations of 2 replicates are reported.

EILV/CHIKV antigen was assessed as a diagnostic reagent in a capture ELISA format. IgM- and IgG-capture ELISAs were performed with acute serum samples collected 8 days post-fever onset from patients with RT-PCR-confirmed CHIKV infection. Samples positive for DENV or VEEV (IAB subtype) but negative for CHIKV by HI were utilized as negative controls. Serum samples were diluted from 1:50 to 1:51,200 and added to IgM- or IgG-capture plates followed by the addition of EILV/CHIKV. Antigen was detected with mouse CHK-175 mAb. Anti-CHIKV IgM was readily detected with OD values ranging from 0.23–3.0, whereas the negative controls yielded a mean OD value of 0.09 (Fig 2A). Signal-to-noise ratios ranged from 2–45:1 for all dilutions except 1:51,200. Anti-CHIKV IgG was also readily detected with OD values ranging from 1.6–2.6, with negative controls yielding a mean OD value of 0.09 (Fig 2B). Signal-to-noise ratios ranged from 24–39:1. No cross-reactivity was observed in either IgM- or IgG-capture ELISAs with anti-VEEV or-DENV human serum.

Next, we compared the IgM- and IgG-capture ELISAs utilizing EILV/CHIKV antigen to commercial human IgM- or IgG-capture ELISA Kits (Abcam). A panel of human serum samples from suspected CHIKV-infected individuals in Mexico was screened by PRNT_50_ and 32 positive samples were selected for IgM ELISAs. A PRNT_50_ was utilized, instead of PRNT_80_, to increase the dynamic range of the assay to allow for more sensitive detection of anti-CHIKV antibodies in the acute phase of disease. For IgG ELISAs, 32 PRNT_80_-positive samples from Bangladesh, collected from healthy individuals, were selected. Serum samples, 4 positive and 8 negative, used above were selected as controls for both IgM and IgG ELISAs. The negative controls were confirmed CHIKV-negative by HI, and some were positive for either DENV or VEEV by HI. To determine a cut-off value for the IgM and IgG ELISAs, 42 human serum samples negative by CHIKV PRNT_80_ were utilized (S1 Table). The OD values ranged from 0.06–0.16 and 0.08–0.12 for IgM and IgG ELISAs, respectively, and cut-off values of 0.18 and 0.13 were determined as 5 standard deviations above the mean absorbance value. For the Abcam IgM and IgG kits, positive samples were determined as per the manufacturer’s protocol.

**Fig 3:**
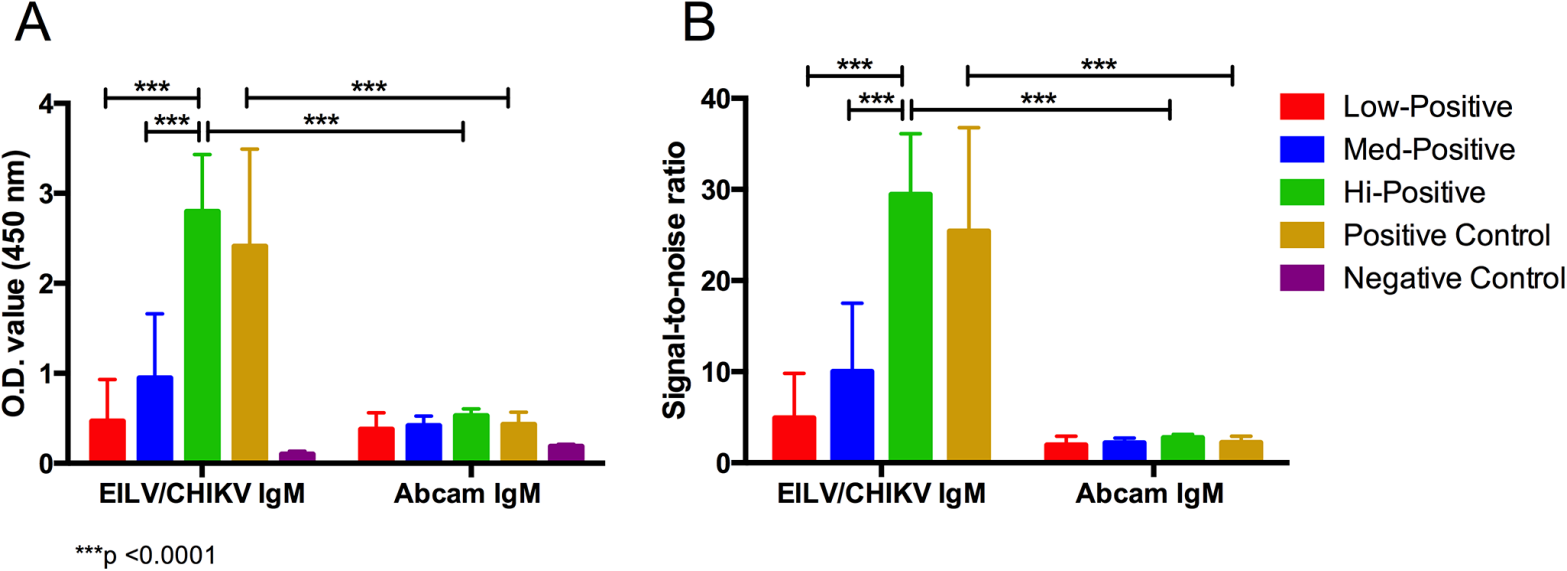
Comparison of EILV/CHIKV and Abcam human anti-CHIKV IgM-capture ELISA methods. (A) raw OD values or (B) signal-to-noise ratios (calculated by dividing positive sample OD values by mean negative control OD value) produced by samples of varying degrees of seropositivity were compared. Low-positive samples had PRNT_50_ ≤80 (n = 4), medium-positive samples had PRNT_50_ 160–320 (n = 4), and high-positive samples had PRNT_50_ >640 (n = 24). Mean and standard deviations are reported. A 2-way ANOVA with Tukey’s test was used to compare absorbance values and signal-to-noise ratios within each ELISA method. 2-way ANOVA with Bonferroni’s test was used to compare absorbance values and signal-to-noise ratios between each ELISA method.

The EILV/CHIKV IgM ELISA detected all 32 PRNT_50_-positive serum samples (100% sensitivity), whereas the Abcam kit yielded positive results for only 13 (41% sensitivity), 11 of which had PRNT_50_ titers >640 (Table 1). An additional 12 of the 32 samples were inconclusive as per instructions provided with the Abcam kit. The 24 samples with PRNT_50_ >640 yielded the highest OD values in EILV/CHIKV IgM ELISA ranging from 1.0–3.3, and signal-to-noise ratios were 11–36:1. In contrast, the OD values of 11 samples positive with the Abcam kit ranged from 0.54–0.7, with signal-to-noise ratios of 2.7–3.5:1. Additionally, the EILV/CHIKV IgM ELISA distinguished between samples with PRNT_50_ > 640 versus ≤ 320, whereas no differences could be discerned with the Abcam kit, which detected only 2 of 8 samples with PRNT_50_ ≤ 320 (Fig 3A and 3B).

**Table 1:** Comparative IgM ELISA results using EILV/CHIKV and Abcam methods.

Sample	Days after onset of symptoms	PRNT_50_	EILV/CHIKV IgM mean OD value	Abcam IgM mean OD value	EILV/CHIKV signal-to-noise ratio	Abcam signal-to-noise ratio	EILV/CHIKV IgM diagnosis	Abcam IgM diagnosis
SW1214	NA	<20	0.07	0.19	NA	NA	-	-
358010P	NA	<20	0.08	0.23	NA	NA	-	-
JL07301	NA	<20	0.07	0.18	NA	NA	-	-
CK08300	NA	<20	0.08	0.2	NA	NA	-	-
NR09270	NA	<20	0.1	0.22	NA	NA	-	-
5343–12	NA	<20	0.16	0.17	NA	NA	-	-
5343–14	NA	<20	0.13	0.2	NA	NA	-	-
RT09270	NA	<20	0.07	0.17	NA	NA	-	-
CH-0027	3	<20*	0.21	0.37	2	2	+	-
CH-0010	3	<20†	0.21	0.21	2	1	+	-
LI-0007	4	80	0.29	0.31	3	2	+	-
CH-0024	5	80	1.16	0.64	12	3	+	+
LI-0003	3	160	0.2	0.32	2	2	+	-
TA-0001	4	320	1.83	0.56	19	3	+	+
LI-0004	6	320	1.18	0.45	12	2	+	?
LI-0027	10	320	0.6	0.37	6	2	+	-
LI-0023	1	>640	2.78	0.45	29	2	+	?
CH-0068	4	>640	2.14	0.49	23	3	+	?
LI-0021	4	>640	3.28	0.66	35	3	+	+
LI-0002	5	>640	1.79	0.52	19	3	+	?
LI-0030	6	>640	3.23	0.6	34	3	+	+
CH-0013	6	>640	3.28	0.55	35	3	+	+
CH-0007	6	>640	3.31	0.55	35	3	+	+
CH-0069	7	>640	2.32	0.39	24	2	+	-
CH-0043	7	>640	2.65	0.53	28	3	+	?
CH-0042	8	>640	1.03	0.41	11	2	+	-
CH-0066	8	>640	2.11	0.53	22	3	+	?
LI-0025	9	>640	2.69	0.53	28	3	+	?
CH-0036	10	>640	2.8	0.54	29	3	+	+
LI-0015	10	>640	3.23	0.55	34	3	+	+
CH-0062	10	>640	3.25	0.57	34	3	+	+
CH-0049	11	>640	3.23	0.48	34	2	+	?
LI-0017	13	>640	1.72	0.47	18	2	+	?
CH-0057	13	>640	3.24	0.52	34	3	+	?
CH-0050	13	>640	3.31	0.53	35	3	+	?
CH-0063	14	>640	3.23	0.56	34	3	+	+
CH-0058	17	>640	3.21	0.7	34	4	+	+
CH-0018	20	>640	3.33	0.69	35	4	+	+
CH-0041		>640	2.77	0.47	29	2	+	?
CH-0032		>640	3.21	0.54	34	3	+	+
Pos 1	NA	NT	3.15	0.55	33	3	+	+
Pos 2	NA	NT	2.33	0.35	25	2	+	-
Pos 3	NA	NT	3.26	0.54	34	3	+	+
Pos 4	NA	NT	0.92	0.28	10	1	+	-

* 44% reduction in plaques.

† 37% reduction in plaques.

OD, optical density. EILV, Eilat virus. CHIKV, chikungunya virus.

NA, not applicable. NT, not tested. +, positive result.–, negative result.?, inconclusive result.

**Fig 4:**
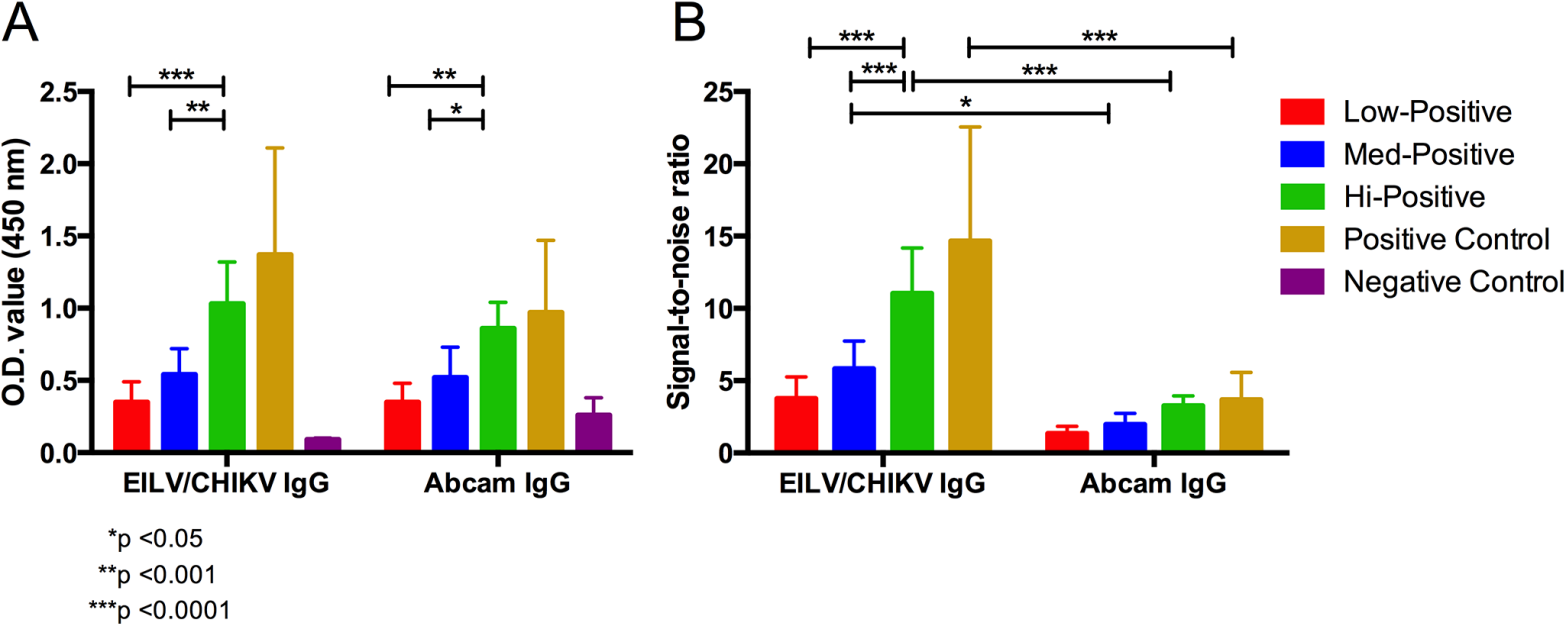
Comparison of EILV/CHIKV and Abcam human anti-CHIKV IgG-capture ELISA methods. (A) raw OD values or (B) signal-to-noise ratios (calculated by dividing positive sample OD values by mean negative control OD value) produced by samples of varying degrees of seropositivity were compared. Low-positive samples had PRNT_80_ ≤80 (n = 10), medium-positive samples had PRNT_80_ 160–320 (n = 10), and high-positive samples had PRNT_80_ >640 (n = 10). Means and standard deviations are reported. A 2-way ANOVA with Tukey’s test was used to compare absorbance values and signal-to-noise ratios within each ELISA method. 2-way ANOVA with Bonferroni’s test was used to compare absorbance values and signal-to-noise ratios between each ELISA method.

The EILV/CHIKV IgG ELISA detected all 32 PRNT_80_-positive serum samples (100% sensitivity), whereas the Abcam kit detected only 23 (72% sensitivity) (Table 2). The Abcam kit detected all 12 samples with PRNT_80_ >640, whereas it could only detect 7 out of 10, and 4 out of 10 samples with PRNT_80_ 160–320, and <80, respectively. Additionally, 3 samples were inconclusive as per the manufacturer’s instructions, and one of the negative controls tested positive with the Abcam kit. The positive samples produced similar OD values in both kits; however differences in OD values of negative controls were observed with OD values ranging from 0.09–0.12 in the EILV/CHIKV ELISA compared to 0.14–0.49 in the Abcam ELISA. Consequently, significant differences in signal-to-noise ratios between EILV/CHIKV and Abcam ELISAs for high (p<0.0001) and medium (p<0.05) PRNT_80_ groups were observed. For high-positive samples with PRNT_80_ >640, EILV/CHIKV ELISA ratios ranged from 7–17:1 and 2–5:1 in the Abcam ELISA (Fig 4A and 4B). For medium-positive samples with PRNT_80_ 160–320, the ratios ranged from 3–8:1 in the EILV/CHIKV ELISA and 1–3:1 in the Abcam ELISA. No differences between EILV/CHIKV and Abcam ELISAs were observed in signal-to-noise ratios of samples with PRNT_80_ <80.

**Table 2:** Comparative IgG ELISA results using EILV/CHIKV and Abcam methods.

Sample	PRNT_80_	EILV/CHIKV IgG mean OD value	Abcam IgG mean OD value	EILV/CHIKV signal-to-noise ratio	Abcam signal-to-noise ratio	EILV/CHIKV IgG diagnosis	Abcam IgG diagnosis
SW1214	NT	0.12	0.14	NA	NA	-	-
358010P	NT	0.09	0.25	NA	NA	-	-
JL07301	NT	0.09	0.49	NA	NA	-	+
CK08300	NT	0.09	0.27	NA	NA	-	-
NR-09270	NT	0.09	0.21	NA	NA	-	-
5343–12	NT	0.08	0.31	NA	NA	-	-
5343–14	NT	0.09	0.30	NA	NA	-	-
RT09270	NT	0.10	0.14	NA	NA	-	-
61106	40	0.68	0.69	7	3	+	+
62901	40	0.39	0.40	4	2	+	+
100103	40	0.26	0.28	3	1	+	-
110303	40	0.23	0.24	2	1	+	-
80305	80	0.21	0.22	2	1	+	-
82202	80	0.41	0.34	4	1	+	?
100305	80	0.40	0.38	4	1	+	+
102204	80	0.34	0.33	4	1	+	?
131201	80	0.33	0.38	4	1	+	+
142201	80	0.25	0.27	3	1	+	-
81108	160	0.62	0.62	7	2	+	+
100306	160	0.48	0.47	5	2	+	+
132201	160	0.58	0.48	6	2	+	+
161101	160	0.31	0.27	3	1	+	-
252101	160	0.32	0.27	3	1	+	-
10502	320	0.36	0.31	4	1	+	?
11102	320	0.51	0.47	5	2	+	+
11303	320	0.76	0.72	8	3	+	+
21204	320	0.73	0.67	8	3	+	+
50503	320	0.77	0.88	8	3	+	+
140403	>640	0.66	0.66	7	3	+	+
141201	>640	1.16	0.92	12	3	+	+
142302	>640	1.31	1.06	14	4	+	+
142303	>640	0.97	0.78	10	3	+	+
150101	>640	0.72	0.60	8	2	+	+
152203	>640	0.68	0.69	7	3	+	+
161203	>640	0.94	0.81	10	3	+	+
170404	>640	0.75	0.76	8	3	+	+
211202	>640	1.23	1.03	13	4	+	+
232103	>640	1.58	1.21	17	5	+	+
362101	>640	1.26	0.94	13	4	+	+
401202	>640	1.10	0.90	12	3	+	+
Pos 1	NT	2.32	1.61	25	6	+	+
Pos 2	NT	0.82	0.40	9	2	+	+
Pos 4	NT	1.58	1.01	17	4	+	+
Pos 5	NT	0.75	0.86	8	3	+	+

OD, optical density. EILV, Eilat virus. CHIKV, chikungunya virus.

NA, not applicable. NT, not tested. +, positive result.–, negative result.?, inconclusive result.

### Stability of EILV/CHIKV antigen

**Fig 5:**
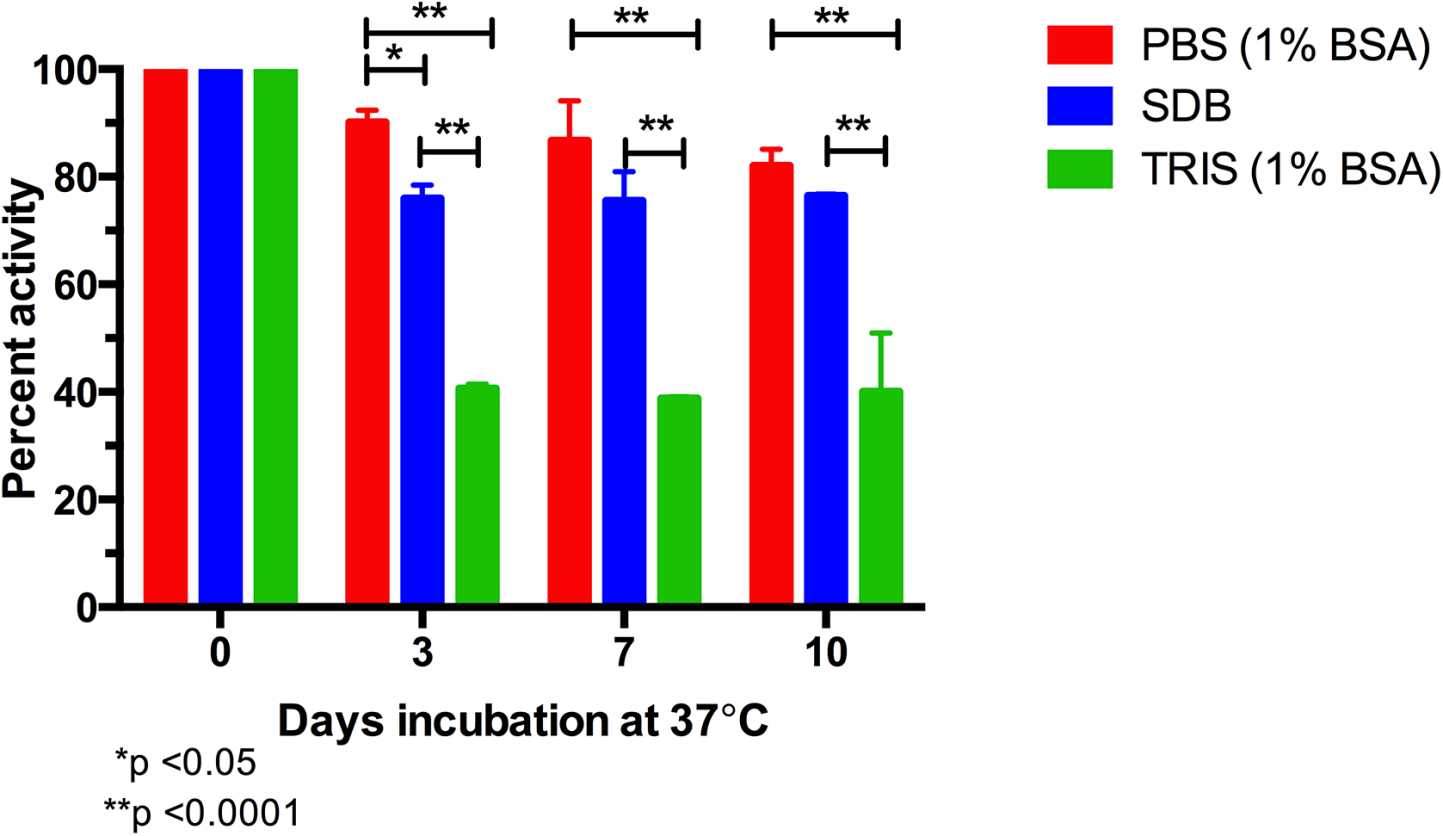
Stability of EILV/CHIKV in different buffers determined by accelerated decay at elevated temperature. Mean and standard deviations of three replicates are reported. A 2-way ANOVA with Tukey’s test was used to compare the effects of different buffer preparations on ELISA activity.

Using the accelerated decay methodology [*34*], EILV/CHIKV was tested for its stability in three different buffers. EILV/CHIKV antigen was relatively stable in IgM-capture ELISA, with mean activity values, relative to initial activity, of 90% at 3 days, 87% at 7 days, and 82% at 10 days at 37°C in PBS-based buffer containing 1% BSA (Fig 5). In contrast, commercial kits generally provide antigen in lyophilized form and, upon reconstitution in buffer, as stated in their protocol, the antigen remains stable for up to 24 hours at 2–8°C. While there was no difference in ELISA activity for EILV/CHIKV stored in PBS (1% BSA) versus SDB at the later time points of 7 and 10 days at 37°C, antigen stored in TRIS (1% BSA) was less stable than that stored in the other two buffers at all time points.

### Safety of EILV/CHIKV *in vivo*

**Fig 6:**
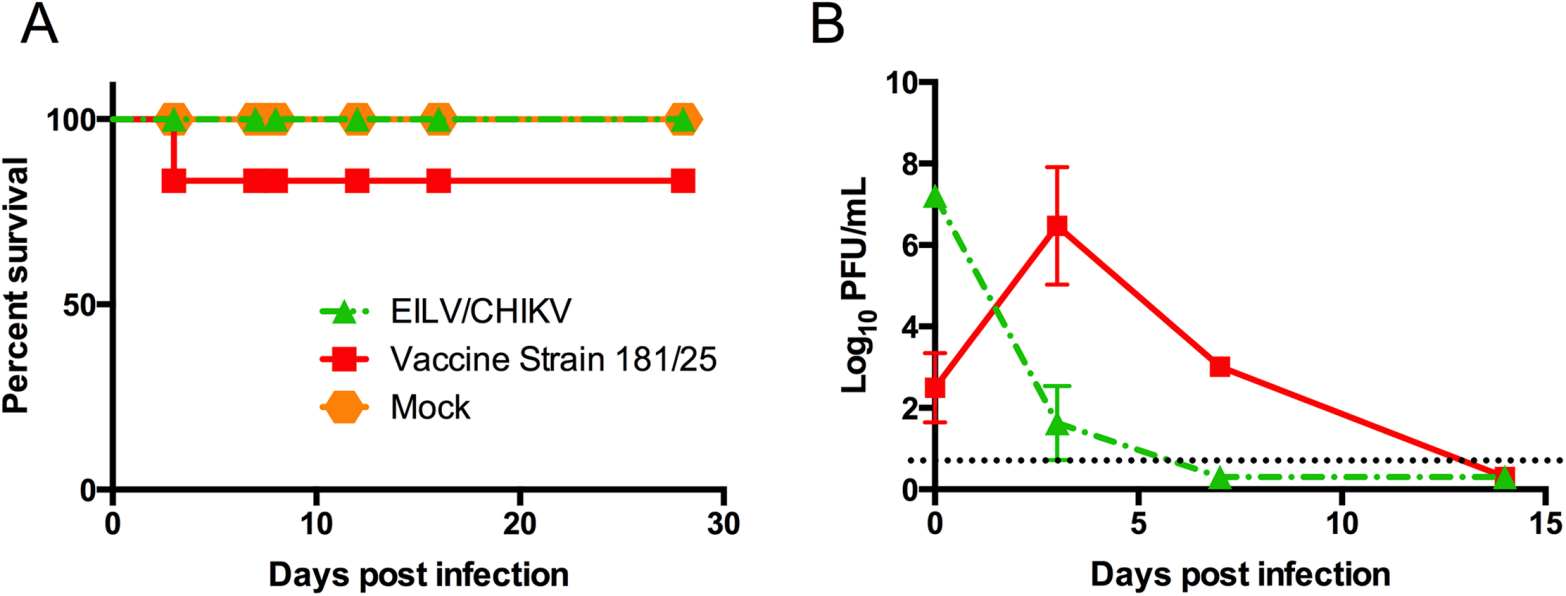
Safety of EILV/CHIKV in the newborn mouse model of neurovirulence. (A) Survival of infant mice infected IC with EILV/CHIKV, live-attenuated vaccine strain 181/25, or PBS. (B) Replication of EILV/CHIKV or strain 181/25 in newborn mouse brain tissue.

Infant mice inoculated IC with 8 log_10_PFU of EILV/CHIKV showed no signs of disease while those infected with a 10,000-fold lower dose of live-attenuated vaccine strain 181/25 did show signs of disease, including hind limb paralysis, hunched posture, and ruffled fur (Fig 6A). In terms of replication, infectious titer of EILV/CHIKV dropped from a mean titer of 1.7X10^7^ PFU/mL at day 0 to 1.3X10^2^ PFU/mL at day 3, and could not be detected on day 7. As expected, positive control strain 181/25 replicated from a mean titer of 9.3X10^2^ PFU/mL on day 0 to 3.1X10^7^ PFU/mL on day 3 (Fig 6B).

## Discussion

Our results indicate that EILV/CHIKV serves as a cost effective ELISA antigen for serological detection of CHIKV infection. In mosquito cells, this chimera replicates to exceptionally high titers (10^10^ PFU/mL), including in serum-free medium. Using a highly sensitive, newborn mouse model, we have demonstrated the replication-incompetence and safety of EILV/CHIKV following IC inoculation. The safety characteristics of EILV/CHIKV eliminate the need for high-level biosafety containment facilities for antigen production, and chemical or physical inactivation, thus maximally preserving native antigens. When applied either in capture- or indirect-ELISA formats using control MIAFs and acute and convalescent human serum samples from the Caribbean and Bangladesh, respectively, we determined that the EILV/CHIKV-based assay provides extremely sensitive indication of infection, outperforming traditional antigens with very high signal-to-noise ratios.

While there are no licensed antiviral treatments available for CHIKV infection, early diagnosis is nevertheless important. Currently, patients lacking a laboratory-confirmed diagnosis are often empirically treated with antibiotics or antimalarials due to similar clinical presentations [*40*]. This can contribute to antimicrobial resistance and unnecessary side effects. In terms of surveillance, early and simple detection of CHIKV infection in resource-poor regions can better inform vector control measures, limiting epidemic spread of disease. Also, distinguishing dengue, which can be life threatening, from CHIKV infection can be important for case management.

To detect recent CHIKV infections, IgM ELISAs are effective due to the prompt production following infection (usually within 4–7 days) of virus-specific IgM [*16*]. Additionally, IgM tends to be less cross-reactive among alphaviruses than IgG, resulting in high specificity [*36*]. Although RT-PCR is effective for diagnosing CHIKF soon after infection, its utility diminishes with time after the onset of signs and symptoms. Typically, by day 5 after symptoms appear, viral RNA levels in serum or plasma fall below detection limits [*16*].

Several commercial kits are available for the detection of anti-CHIKV IgM antibodies. However, their lack of specificity and sensitivity limits their clinical use [*36*]. Current PAHO, World Health Organization, and CDC guidelines recommend that in-house IgM ELISAs be implemented for accurate CHIKF diagnostics, and the use of whole virus antigen as opposed to recombinant subunits is recommended [*33*]. Of the commercial kits, Abcam’s Anti-CHIKV IgM Human ELISA kit is reported to produce comparable results to the CDC IgM ELISA [*33*]. In our comparative analysis, the EILV/CHIKV IgM ELISA demonstrated improved sensitivity with a maximum signal-to-noise ratio of 35:1 compared to a maximum ratio of 4:1 for the Abcam kit. Overall, the signal-to-noise ratios of the EILV/CHIKV IgM ELISAs were consistently higher than those produced by the Abcam kit. In laboratories that lack access to plate readers to quantify optical density, high signal-to-noise ratios can aid in the differentiation of positive and negative samples by simple visual observation.

Of the 32 positive controls that we tested, 6 were collected between 4 and 5 days after the onset of symptoms. Of those, 5 had signal-to-noise ratios of 12–35:1, allowing for confident identification of CHIKV IgM-positivity without the need for a plate reader. Samples collected less than 4 days after onset would require the use of a plate reader. For reliable detection of acute CHIKV-infection, EILV/CHIKV IgM-capture ELISA can be used alone, or in conjunction with, reverse transcriptase-PCR testing.

Traditional approaches to antigen production for alphaviruses require infection of cell cultures or mouse brains in biosafety facilities, followed by time-consuming and expensive procedures to purify, concentrate, and inactivate the virus [*37*]. Manipulations such as these also have the potential to adversely affect the antigen, resulting in loss of epitopes. For instance, our mouse anti-CHIKV mAb readily detected EILV/CHIKV-derived antigen but not that derived from CLA (Fig 1). Recently, it was reported that complete CHIKV inactivation while retaining acceptable ELISA activity requires gamma-irradiation, followed by β-propiolactone-inactivation [*38*]. Furthermore, such preparations are unstable at 4°C, requiring that the antigen be lyophilized for storage longer than 24 hours [*38*]. As a result, the cost of antigen production from CHIKV cultures can be high and many of the commercially available ELISA kits are prohibitively expensive for clinical laboratories in developing countries endemic for alphaviruses. Recombinant protein antigens, derived from cloning and expressing partial viral genomes, can sometimes be missing critical conformational epitopes or may not fold correctly during production, resulting in serologic reduced sensitivity. Virus-like particle antigens, on the other hand, should contain conformational epitopes, but concentration and purification steps can complicate production [*41*]. Using the EILV chimeric platform, the cost of production can be reduced by eliminating the need for containment, purification, concentration, and inactivation. EILV/CHIKV also displays remarkable stability in liquid buffer, with an estimated shelf life at 4°C of >1 year. These qualities suggest that EILV-based chimeras are superior to traditional inactivated virus antigens for alphavirus diagnostics.

While we have shown the utility of an EILV-based chimera as an ELISA antigen, further testing and evaluation is needed to define parameters for the clear discrimination between positive and negative results. We have shown that our anti-CHIKV IgM and IgG ELISAs can distinguish between human infections caused by CHIKV and VEEV or DENV with a high degree of signal separation. However, because other closely related alphaviruses co-circulate in most if not all CHIKF-endemic regions (e.g. Mayaro virus in South America [*39*]), more extensive testing is needed to rule out false positives due to cross-reactivity with antibodies elicited by other alphaviruses, and thus determine the specificity of this assay. Also, while we have performed accelerated-decay studies to evaluate the stability of EILV/CHIKV in liquid storage, real-time stability studies need to be performed to determine the long-term stability of the antigen at 4°C.

## Supporting Information

S1 Table Optical density values of serum samples negative by PRNT used to calculate cut-off values for IgM and IgG ELISAs. (DOCX)
