# Supplementary material for: Utilization of an Eilat Virus-Based Chimera for Serological Detection of Chikungunya Infection
Source: PLoS Negl Trop Dis. 2015 Oct 22;9(10):e0004119. doi: 10.1371/journal.pntd.0004119 (PMC4619601; doi:10.1371/journal.pntd.0004119)
Supplement: S1 Table — (DOCX) [file pntd.0004119.s001.docx]

**S1 Table. Optical density values of serum samples negative by PRNT**

| **Sample** | **PRNT_50_** | **EILV/CHIKV IgM OD Value** | **EILV/CHIKV IgG OD Value** |
| --- | --- | --- | --- |
| 61104 | <20 | 0.07 | 0.10 |
| 52401 | <20 | 0.07 | 0.10 |
| 52403 | <20 | 0.08 | 0.09 |
| 80203 | <20 | 0.06 | 0.09 |
| 82301 | <20 | 0.09 | 0.09 |
| 102202 | <20 | 0.08 | 0.08 |
| 132101 | <20 | 0.09 | 0.08 |
| 140406 | <20 | 0.08 | 0.10 |
| 180101 | <20 | 0.08 | 0.09 |
| 191202 | <20 | 0.1 | 0.10 |
| 210205 | <20 | 0.07 | 0.08 |
| 210208 | <20 | 0.07 | 0.09 |
| 210302 | <20 | 0.09 | 0.09 |
| 220401 | <20 | 0.07 | 0.11 |
| 222101 | <20 | 0.09 | 0.09 |
| 230304 | <20 | 0.1 | 0.10 |
| 242206 | <20 | 0.09 | 0.09 |
| 242604 | <20 | 0.09 | 0.10 |
| 252303 | <20 | 0.08 | 0.09 |
| 260104 | <20 | 0.1 | 0.09 |
| 270302 | <20 | 0.13 | 0.10 |
| 272102 | <20 | 0.1 | 0.10 |
| 281101 | <20 | 0.1 | 0.09 |
| 291102 | <20 | 0.08 | 0.10 |
| 291204 | <20 | 0.09 | 0.10 |
| 292104 | <20 | 0.09 | 0.10 |
| 321102 | <20 | 0.09 | 0.09 |
| 322204 | <20 | 0.11 | 0.09 |
| 341103 | <20 | 0.1 | 0.08 |
| 350301 | <20 | 0.09 | 0.08 |
| 352304 | <20 | 0.09 | 0.09 |
| 372208 | <20 | 0.11 | 0.09 |
| 382303 | <20 | 0.09 | 0.09 |
| 402301 | <20 | 0.09 | 0.09 |
| SW1214 | <20 | 0.07 | 0.12 |
| 358010P | <20 | 0.08 | 0.09 |
| JL073012 | <20 | 0.07 | 0.09 |
| CK083005 | <20 | 0.08 | 0.09 |
| NR092702 | <20 | 0.1 | 0.09 |
| 5343-12 | <20 | 0.16 | 0.08 |
| 5343-14 | <20 | 0.13 | 0.09 |
| RT092702 | <20 | 0.07 | 0.10 |

OD, optical density. EILV, Eilat virus. CHIKV, chikungunya virus.
